# A revolution in cervical cancer prevention in Ghana

**DOI:** 10.3332/ecancer.2022.ed123

**Published:** 2022-08-09

**Authors:** Kofi Effah, Ethel Tekpor, Comfort Mawusi Wormenor, Bernard Hayford Atuguba, Isaac Gedzah, Joseph Emmanuel Amuah, Patrick Kafui Akakpo

**Affiliations:** 1 Cervical Cancer Prevention and Training Centre, Catholic Hospital, Battor, PO Box 2, Battor, via Sogakope, Volta Region, Ghana; 2 Department of Obstetrics and Gynaecology, Catholic Hospital, Battor, PO Box 2, Battor, via Sogakope, Volta Region, Ghana; 3 Laboratory, Catholic Hospital, Battor, PO Box 2, Battor, via Sogakope, Volta Region, Ghana; 4 School of Epidemiology and Public Health, Faculty of Medicine, University of Ottawa, 451 Smyth Road (2046), Ottawa, ON K1H 8M5, Canada; 5 Department of Pathology, University of Cape Coast, School of Medical Sciences, Clinical Teaching Center, Private Mail Bag, GN-06035819, Cape Coast, Ghana

**Keywords:** cervical, breast, cancer, screening, Human Papillomavirus, programmes, low resource settings

## Abstract

Though cervical cancer is largely preventable, success depends on sustained screening and treatment of precancer. This is not available in many low resource settings where screening and treatment services are not available due to a lack of government support. Our vision of setting up a comprehensive cervical cancer prevention scheme across Ghana that offers services tailored to fit every patient’s needs, and relies on task shifting has been made possible through the setting up of the Cervical Cancer Prevention and Training Centre (CCPTC) to train and equip middle cadre staff (mostly nurses and midwives) to provide crucial cervical precancer screening and treatment services in many areas of the country that have never seen any such screening activities. To achieve this vision, we have learnt to produce crucial context relevant teaching materials and consumables locally, while adapting simple, readily available social media applications to raise crowd funds to support our work, use these apps to support routine work and to create a network of service providers at various service levels that can rely on each other and assure quality. Our vision has been supported by individuals and organizations that believe in it. They have allowed us to determine our growth and success. By sharing the experiences of the CCPTC we hope to encourage others to set up screening centers in low resource settings.

Current estimates indicate that every year 2,797 women are diagnosed with cervical cancer and 1,699 die from the disease in Ghana. Cervical cancer is the second leading cause of cancer in women in Ghana after breast cancer [1]. While cervical cancer is very preventable with primary prevention (HPV vaccination) and secondary prevention (screening and treatment of precancerous lesions of the cervix), Ghana has no national HPV vaccination programme and less than 3% of eligible women are screened for cervical precancer [1]. Many women therefore present to health institutions with advanced cervical cancer [2].

Another challenge has been the educational system in Ghana. Sadly, generally, emphasis has not been placed on bringing out graduates equipped with practical skills to solve our problems. There is a big difference in theoretical knowledge and practical skills. This is clear in cervical cancer prevention. Most nurses, midwives, Physician Assistants and Medical Doctors (including Specialists) graduate without the skills to screen for cervical precancer and treat precancerous lesions of the cervix to prevent cancer [3].

With these gaps identified, the Cervical Cancer Prevention and Training Centre (CCPTC) was established in 2017 to build human resource capacity across Ghana and in other similar low resource settings. Catholic Hospital, Battor was started in 1957 by four German nuns. Other nuns came in later. The last two nuns left in 2012. The hospital is part of the Christian Health Association of Ghana (CHAG) under the Ministry of Health Ghana, which is in charge of the national health policy. The Ghanaian management team in charge of the hospital has tried to be self-reliant as much as possible. The setting up of the CCPTC was immensely supported by the Management team of the Catholic Hospital, Battor. This included the Medical Superintendent, Administrator, and the Nurse Manager. Their involvement ensured that administrative support and human resources were readily available to run the CCPTC while ensuring some semi autonomy. It is essential that such initiatives court and receive such managerial support to run successfully and achieve the vision. [4]. A ‘revolution’ in cervical cancer prevention had begun in Ghana [5].

The vision was to create a sustainable system not dependent on external funding. This was not easy, but we knew it is possible. A lot of work was done. Two modules were developed for the training. Module 1 includes practical and theoretical sessions that equip trainees with the knowledge and skills to set up a screening unit and screen for cervical precancer. Module 2 equips trainees with the knowledge and skills to treat precancerous lesions of the cervix with thermal coagulation and cryotherapy. These modules are higher than the traditional training in Visual Inspection with Acetic Acid (VIA) and involves some training in Basic Colposcopy [6, 7]. In both modules, emphasis is placed on practical skills to ensure that trainees can practise wherever they find themselves. To ensure trainees have adequate practise during their training, outreaches are organized, and trainees are involved in the daily activities at the CCPTC. Included in the cost of training for each trainee is the cost of screening 15 women with VIA. This ensures there are women available for screening during the training period even if the women cannot pay themselves. With a class size of up to 14 trainees, it means up to 210 women can be screened ‘for free.’ The CCPTC uses this to organise outreaches in communities in and around the North Tongu District (where the CCPTC is situated). This ensures that women in the communities who would otherwise not have been able to pay, get screened. This is sustainable because as long as trainees pay to come for the training, they contribute to screening women. Trainees also benefit as they get hands-on experience during these outreaches. Nurses and Physician trainees are usually sponsored by their institutions to come for the training. The CCPTC currently has six nurses involved in the screening, treatment of precancer and training. There are monthly quality assurance meetings which involves a gynaecologist and the nurses at the centre. Images from all screen positives are reviewed during these meetings.

To equip the center to run, many approaches had to be adopted to raise funds to support the purchase of basic but necessary equipment to be able to perform at the highest level. We employed crowd funding to purchase initial equipment for HPV testing and a thermal coagulator for treatment of precancerous lesions of the cervix. Our experience is published in a manuscript [8]. Many individuals and organizations acknowledged in this editorial supported the CCPTC by donating key equipment and consumables, cash or by supporting the training of trainees and outreaches [9,10,11,12,13,14]. Many of these donors personally visited the CCPTC. The belief in the vision of these donors led to sponsorship of the CCPTC’s own pre-determined programmes without the imposition of unnecessary conditions and terms that could derail the smooth running of services at the CCPTC or change its vision. To succeed, many programmes in poor resource settings need to carefully choose their partners and donors to ensure that they are not forced to follow the vision of their donors and partners. In our experience, the best partners are those who support our vision and mission in diverse ways determined by our needs. An important point is that our hospital can buy many of the equipment that we raise funds for, but when this is done, it means that the cost must be transferred to clients/ patients. Poor women in remote communities can then not afford these services. If we raise funds for these or get donations for these, clients can pay only a token for maintenance and replacement of the equipment in the long term. Even those who cannot afford these low costs can get the service for free at times [8].

While there have been challenges, including the Covid-19 pandemic disrupting our training programme [15], we have been highly successful and made progress with some great innovations. The CCPTC launched an app for screening for cervical precancer in June 2018 making it easy for health workers anywhere to use our algorithms [16, Appendix]. Many items needed for cervical cancer screening like Acetic acid (kitchen vinegar), Lugol’s iodine, Trichloroacetic acid (for treatment of warts), Monsel’s solution (to control bleeding after a biopsy) that were imported are now produced in Ghana for our work because we now have the numbers that need them to make local production financially attractive.

The CCPTC has so far trained 271 health workers (nurses, midwives, physician assistants, medical doctors) in 184 institutions in 119 (out of the 216) districts in all 16 Regions in Ghana. We have trained two nurses in Liberia and a medical doctor from Sierra Leone. More health workers across Ghana continue to be trained at the CCPTC. In 2021, we involved the Department of Surgery in Catholic Hospital, Battor and included early breast cancer detection with clinical breast examination in our training programme. This means apart from the training in cervical cancer prevention, our trainees are now taught early detection of breast cancer [17]. Our alumni are now picking up breast cancer early across the country. These have offered a great opportunity to have a cervical cancer prevention programme combined with an early breast cancer detection programme across Ghana using simple available tools and human resource. Our focus on task shifting means that many of our trainees are nurses who practise at primary health care facilities in the communities where screening is key and treatment of detected per-cancer is crucial. By equipping trainees with essential skills for screening and treatment of precancer, many patients are screened and treated.

Each cohort of trainees (up to 14), is put on a social media platform (WhatsApp) with the trainers before the group arrives in Battor. This enables the trainers to share teaching materials before and during the period of the training. Once they complete the training, they are added to a bigger WhatsApp platform that has all the alumni from across the country. This ensures continuous education. Alumni from across the country share activities they are involved in. There is networking, and the platform makes it easy to refer women anywhere in Ghana to the closest facility where they can get screened. It also facilitates referral of patients with cervical and breast lesions to institutions where they can get the needed care when cases are beyond the expertise of our trainees. By relying on this concept of a ‘hub and spokes’, many spokes (primary care facilities) are linked to few larger more advanced centers like the CCPTC for continuous support. In practise, alumni in institutions that have medical doctors trained in breast surgery refer women with breast lumps to these medical doctors for further management (biopsies, lumpectomies etc.) or to the nearest secondary or tertiary care facility. Women with cervical lesions suspicious of cancer are referred to centres with medical doctors who can biopsy these lesions. Women with early cervical cancer are referred to institutions where they can have radical hysterectomy or chemoradiation. There are 3 centres in Ghana that offer chemoradiation - one in Kumasi, and two in Accra, the capital city. Alumni who do not know exactly where (the closest institution) to refer women with cervical (pre)cancer can discuss it on the WhatsApp platform. Other alumni respond and direct them. In November 2017, the CCPTC adopted a model of screening involving HPV DNA testing/cytology and a visual inspection method (VIA or mobile colposcopy) done at the same setting. This has reduced the number of women lost to follow up as many women do not have to be recalled for follow up with colposcopy (or VIA) after a positive test for high-risk HPV or cytology. It is simple and cheap (not requiring specula and gloves on another visit, the only requirements - swabs and acetic acid are cheap), and it also gives opportunity to our trainees to have practical experience in performing VIA and in the use of the mobile colposcope.

mPharma is a technology-based healthcare company aimed at making quality health accessible and affordable and to have an Africa in good health. It has offices in 10 countries with the headquarters in Accra, Ghana. In September 2021, mPharma launched the 10,000 Women Initiative [18] which aimed to give free cervical cancer screening with HPV DNA testing to 10,000 women in Ghana and Nigeria (6,000 in Ghana and 4,000 in Nigeria). The emphasis was on women who could otherwise not afford HPV DNA testing. CCPTC led this project in Ghana offering screening for women in all the 16 Regions of Ghana using the alumni across the country. Screening was done with brushes taken for HPV DNA testing by the health workers, and Visual Inspection with Acetic acid (VIA) done at the same setting. A small proportion of women had self-sampling with Evalyn brushes. The brushes were sent to the laboratory at the CCPTC for the HPV DNA testing. Women who had precancerous lesions of the cervix were either treated on site with thermal coagulation or referred to the CCPTC or the nearest facility where treatment could be done. The precancerous lesions of the cervix that did not qualify for treatment with thermal coagulation were treated with Loop Electrosurgical Excision Procedure (LEEP). Nurses have not been trained to perform LEEP and mostly work in primary care settings that lack the equipment needed to perform LEEPs. The aim of the CCPTC is to make cervical cancer prevention services available to women across Ghana using simple tools. As the coverage of cervical screening increases with an increase in the number of precancerous lesions of the cervix not amenable to treatment by ablation, a decision will have to be taken if there is a need for a change in policy for nurses to be trained to perform LEEPs, especially if there are not enough medical doctors to perform the LEEPs. This will also require logistic support. The current policy in Ghana is that LEEPs have to be performed by medical doctors mostly at secondary and tertiary care facilities. Alumni who had been trained to perform clinical breast examination at the CCPTC also offered free clinical breast examination to the women. The mPharma 10,000 Women Initiative has demonstrated that a national cervical precancer screening with HPV DNA testing (and breast cancer screening using trained nurses and midwives to perform clinical breast examination) is feasible.

Our staff at the CCPTC have worked hard training all cadres of health workers. They have screened women in all corners in the country including screening head porters (kayayei), commercial sex workers, nuns and prisoners [19]. Our alumni across the country are also screening women in communities that would otherwise not have seen this for many years. Our main limitation has been that women in Ghana have to pay from their pockets to get screened so many women in remote communities cannot pay for screening. To ensure that as many women as possible get the opportunity to get screened, our approach has been to offer a comprehensive service that includes all methods of cervical precancer screening at various facility levels. These methods include VIA, mobile colposcopy with the Enhanced Visual Assessment (EVA) system, cytology (PAP smear) and HPV DNA testing, with different costs, VIA being the cheapest (approximately $2.5) and HPV DNA testing being the most expensive (approximately $19). The CCPTC has screened over 10,000 women in the last 5 years. The mPharma 10,000 Women Campaign gave additional 5,000 women across Ghana the opportunity to be screened without the women having to pay. We are yet to have an evaluation of the number of women screened across Ghana by alumni of the CCPTC.

The CCPTC has developed data collection forms that are completed by trained nurses who do the screening and treatment of cervical precancer. The collected data is then entered into a secure REDCap database. As part of monitoring and evaluation, the CCPTC is working with a partner institution to objectively evaluate the impact made since the inception despite the anecdotal evidence.

The CCPTC celebrated its 5th anniversary on Tuesday, May 31, 2022. It has been a journey full of lessons worth sharing, especially with other low (middle) income countries. Many important lessons emerge from the CCPTC experience like the central importance of self-reliance, the development of local educational material as opposed to retrofitting approaches that may have been successful in other environments that are inappropriate for Ghana, novel funding schemes to underwrite some of the expenses of the programme, providing free screening for women who cannot afford the costs, and decreasing dependency on external donors, in-country production of some of the materials needed to sustain the programme, the use of crowd funding to raise funds to purchase equipment, task-shifting of procedures from doctors to nurses, the use of a WhatsApp social platform for distance consultation and continuing medical education of trainees, the liberal use of contextually appropriate technology including the use of Apps and the use of multiple screening modalities. We share our experiences because we believe there are lessons for other settings like ours in Ghana and across the world [20, 21].

## Conclusion

Setting up a comprehensive, sustainable cervical precancer screening program in a poor resource setting can be achieved through a model that relies on task shifting to committed personnel who are available in the communities where screening is carried out and are enthusiastic about their new role. Sustained success is possible through innovative and judicious use of available local resources including appropriate adoption and adaption of existing and new technologies. The support of local managers and administrators as well as partners who believe in the vision and are committed to allowing it to grow is crucial. This has been demonstrated by the CCPTC’s program in Battor, which continues to evolve in response to new knowledge and technologies.

## Funding

The authors did not receive any funding for this paper.

## Conflicts of interest

Dr. Kofi Effah is the head of the CCPTC, Ms Ethel Tekpor is the Nurse in Charge of the CCPTC. Dr. Bernard Hayford Atuguba is the Medical Superintendent of Catholic Hospital, Battor. Mr. Isaac Gedzah is the head of the Laboratory in Catholic Hospital, Battor. Ms. Comfort Mawusi Wormenor is a nurse at the CCPTC. Dr. Joseph Emmanuel Amuah and Dr. Kafui Akakpo are affiliated to the CCPTC, involved in research. They all have nothing to declare.

## Figures and Tables

**Figure:**
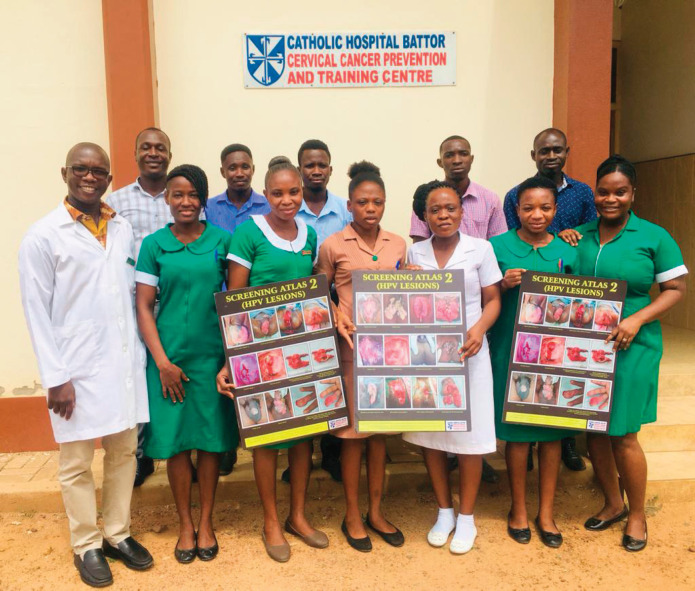
Staff of CCPTC

**Figure:**
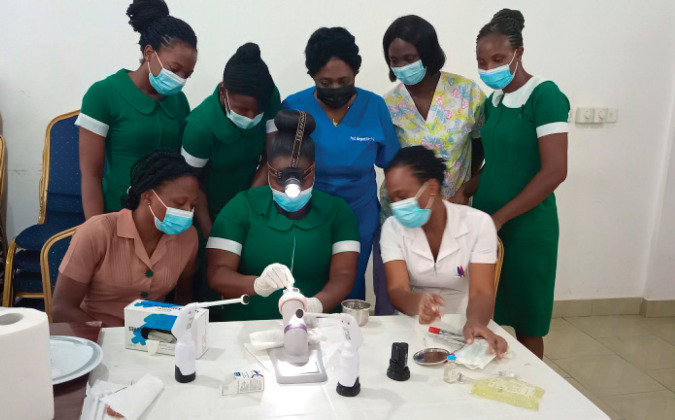
Training in the use of the thermal coagulator for treatment of cervical precancer

**Figure:**
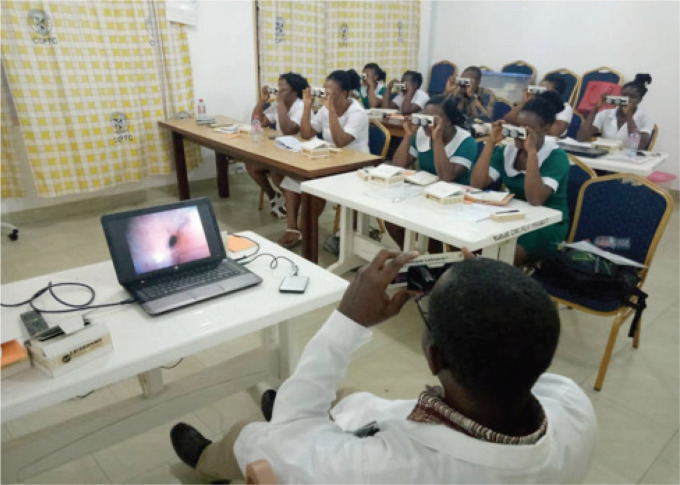
Colposcopy simulation at lectures.

**Figure:**
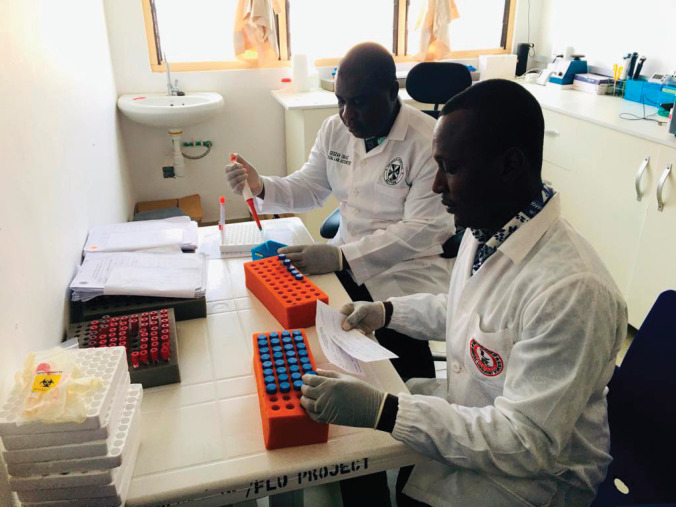
Laboratory staff running HPV DNA tests.

## Appendix 1

CCPTC’s two mobile apps:

1. Application software for cervical precancer screening with mobile colposcopy (Enhanced Visual Assessment system) https://youtu.be/JrFm9A96MQw
2. Application software for treatment of cervical precancer with ablation (Cryotherapy, Thermal coagulation) https://youtu.be/LoDDQeW7-JM

Embedded in the cost of training for Module 1 is the cost of these two apps. Once installed on a mobile phone, a health worker can get access to the CCPTC’s algorithms for screening with mobile colposcopy (or Visual Inspection with Acetic acid) and eligibility criteria for treatment of precancerous lesions of the cervix with ablation (cryotherapy/thermal coagulation). Internet services are not required to use these apps. The apps were developed to give health workers tools for learning and teaching that can aid in decision making even in remote settings and to standardise care of patients.
